# Should We Accept the Epiligament Theory About the Differences in the Healing Potential of the Medial Collateral and the Anterior Cruciate Ligament?

**DOI:** 10.3390/biomedicines13020522

**Published:** 2025-02-19

**Authors:** Georgi P. Georgiev, Lyubomir Gaydarski, Boycho Landzhov

**Affiliations:** 1 Department of Orthopedics and Traumatology, University Hospital Queen Giovanna-ISUL, Medical University of Sofia, 1527 Sofia, Bulgaria; 2 Department of Anatomy, Histology and Embryology, Medical University of Sofia, 1431 Sofia, Bulgaria; lgaidarsky@gmail.com (L.G.); landzhov_medac@abv.bg (B.L.)

**Keywords:** epiligament, medial collateral ligament, anterior cruciate ligament, injury, human

## Abstract

The epiligament (EL), described in 1990 as a connective tissue layer distinguishable from the ligament proper, has only recently gained recognition for its critical role in ligament function and repair. Previously overlooked, the EL is now understood to be a dynamic structure, particularly in the context of medial collateral ligament (MCL) healing. Rat model studies demonstrate that the EL actively contributes to ligament repair by serving as a source of cells and blood vessels, findings later corroborated in human studies. The EL’s role in spontaneous MCL healing highlights its importance, raising the question of whether differences in EL morphology and activity contribute to the poor healing capacity of the anterior cruciate ligament (ACL). Comparative studies reveal significant disparities in EL cellularity and activity between the ACL and MCL. The EL of the MCL is hypercellular, with robust expression markers like α-smooth muscle actin (α-SMA) and collagen types III and V, essential for tissue remodeling and structural integrity. Conversely, the ACL’s EL is less vascularized and exhibits weaker expression of these markers. While vascular endothelial growth factor (VEGF) promotes angiogenesis, its effectiveness is limited in the ACL due to restricted vascularization. Similarly, CD34, a progenitor cell marker, is more prominently expressed in the MCL’s EL, further supporting its superior healing potential. These findings suggest that the EL’s distinct structural and functional attributes are key determinants of ligament healing. Targeting the EL’s regenerative properties offers a promising therapeutic strategy, particularly for improving ACL repair outcomes. Further research is necessary to validate and expand these findings.

## 1. Introduction

Ligamentous knee injuries are common in sports and can occur through a variety of mechanisms. If left untreated, these injuries may lead to persistent instability, progressive damage to the menisci and articular cartilage, and early-onset osteoarthritis [1,2]. The MCL is the most frequently injured knee ligament, especially in sports like soccer, skiing, and ice hockey. These injuries typically result from a direct valgus force to the knee or during cutting maneuvers when an athlete plants their foot and suddenly changes direction or speed [3,4,5,6,7,8,9]. MCL injuries have an incidence rate ranging from 0.24 to 7.3 per 1000 people, with a male-to-female ratio of 2:1 [10,11]. The MCL accounts for up to 40% of all knee ligament injuries [12] and 7.9% of all knee injuries in athletes [13].

The ACL is also one of the most commonly injured knee structures [14], with ACL injuries occurring at 68.6 cases per 100,000 people in the United States [15,16]. These injuries usually happen during sports activities involving twisting and cutting [14,17]. ACL injuries are a significant risk factor for developing osteoarthritis [18]. Over time, the incidence of ACL injuries has steadily increased, making them a significant concern in orthopedic practice. The ACL has a limited capacity for spontaneous healing, unlike the MCL [4,19]. Numerous studies using animal models have explored the healing potential of knee ligaments, offering various explanations for the differences in healing between the two [20,21,22]. Unlike the ACL, the MCL is an extra-articular ligament that can heal naturally, showing considerable recovery potential [23]. This healing capacity has been demonstrated in histological studies, clinical observations, and radiological assessments [20,23]. However, surgical alignment, immobilization, and early protected range of motion can influence healing [23]. Zhang et al. [24] found that stem cells derived from the ACL show inherent differences compared to those from the MCL, which could help explain the disparity in healing abilities between the two ligaments. Despite extensive research, several key questions remain unanswered [24].

This commentary draws attention to a different theory regarding the MCL’s healing and the ACL’s failure. Why revisit these ligaments? Are their biology and morphology not already well understood? While much has been learned about the differing healing capacities of the ACL and MCL, and various theories attempt to explain these differences, the existing knowledge remains incomplete [1,2,3,4,5,6,7,8,9,10,11,12,13,14,15,16,17,18,21,22,23,24]. A recent proposal highlights the potential role of the epiligament (EL) in MCL healing, sparking interest [19,20,25,26,27,28,29,30]. However, is the EL a well-studied structure? Unlike previous studies that have examined ligament healing exclusively in either animal models or human subjects, the research group led by Georgiev conducted a comparative analysis of the EL in a rat model and humans. This approach enabled them to draw more robust conclusions regarding the role of the EL in ligament healing. Moreover, could this theory also shed light on the limited healing capacity of the ACL?

## 2. Main Text

Although the treatment of ligament injuries has significantly improved over the years, many questions remain regarding the completeness of ligament healing [31]. Various studies have shown that while the MCL typically heals spontaneously, it is not fully restored [19,25]. It is well established that the ligament healing process depends on several factors, including anatomical location, associated injuries, and different treatment modalities. These modalities include tissue engineering, nonsteroidal anti-inflammatory drugs, local corticosteroids, hyperbaric oxygen therapy, growth factors, ultrasound or electrical stimulation, laser therapy, and gene therapy [19,25,31,32,33].

The EL was first identified by Bray et al. [34] as a thin layer of connective tissue surrounding the ligament. Chowdhury et al. [35] examined the EL of the MCL in rabbits and concluded that the tissue comprises collagen fiber bundles, three distinct types of cells (spinous-shaped, cuboidal-shaped, and fat cells), and a neurovascular network that periodically extends into the MCL [35]. More recently, Georgiev et al. [20] described the MCL EL as a source of fibroblasts, progenitor cells, and blood vessels that migrate through the endoligament into the ligament body. These authors emphasize its critical role in ligament function and healing. Nonetheless, how was this role demonstrated?

Studies by Georgiev et al. [20,25] using MCL injury models have shown that fibroblasts within the EL are not static; instead, they actively synthesize various collagen types, matrix metalloproteinases, decorin, fibronectin, and fibromodulin. These molecules are widely recognized as key contributors to ligament degradation, proliferation, and remodeling following trauma [20,25].

Following investigations of the EL in rats, a study was conducted to examine this tissue in humans. To explore the proposed role of the EL and address the question of why the ACL fails to heal effectively, Georgiev et al. [26] analyzed the morphology of the mid-substance of the EL in the human MCL and ACL. They formulated a theory regarding ACL healing failure by detailing the EL’s morphology and comparing its characteristics between these two ligaments.

What is currently known about ACL healing failure? Georgiev et al. [26] reviewed the existing theories in the literature, highlighting key factors contributing to the ACL’s limited healing potential. Differences in the ultrastructural characteristics of connective tissue cells between the MCL and ACL have been observed [36]. Additionally, variations in fibroblast proliferation potential [22,37] and elevated nitric oxide levels in the ACL, which inhibit collagen and proteoglycan synthesis, have been identified [21]. The MCL demonstrates a superior capacity to enhance blood flow and angiogenesis following injury [38]. Furthermore, stem cells exhibit ligament-specific properties that differ between the MCL and ACL [24,39], and differential expression of matrix metalloproteinases (MMP-2, -9, and -13) also play a significant role [20,40].

The ACL’s poor healing is also attributed to the inability of cells and blood vessels to bridge the ruptured ends effectively and the inadequate wound filling in the intra-articular environment [41]. The intra-articular ACL is exposed to synovial fluid, inhibiting fibroblast activity [42]. Additionally, plasmin in the synovial fluid degrades the fibrin clot, further impeding healing [43].

What does the EL theory propose, and what evidence supports it compared to previous theories? Table 1 summarizes the current understanding of ACL healing failure and contrasts it with the EL-based theory, offering fresh insights into the differences in healing potential.

These findings evaluate current theories and lay the groundwork for a new perspective on the limited healing capacity of the ACL. Research indicates that cell density is significantly higher in the EL of the MCL compared to the ACL [26,30]. Additionally, the EL of the MCL exhibits a stronger expression of key collagens than that of the ACL. Based on these observations, along with the established roles and functional activity of the EL, a logical explanation for the differing healing potentials of these ligaments has been proposed [26]. The EL theory accounts for the lack of healing observed in middle-third ACL injuries. However, how was the EL theory validated?

Firstly, Georgiev et al. [19,20,25,26,27,30] investigated the structural and functional differences between the ELs of the mid substance of ACL and MCL in both human and animal models, with a focus on understanding the superior healing capacity of the MCL. Their findings highlight that the EL of the MCL contains a higher density of fibroblasts, greater collagen type III and V expression, and more robust vascularization compared to the ACL’s EL [19,20,26]. These features enable the MCL to form granulation tissue, promote angiogenesis, and synthesize collagen more effectively during the early stages of healing. In contrast, the EL of the ACL is less cellular and vascularized, which correlates with its limited healing potential [19,20,26]. Immunohistochemical analyses revealed stronger collagen expression and more pronounced healing-related activities in the MCL’s EL compared to the ACL [19,20,26]. These findings suggest that targeting the EL’s cellular and molecular components could enhance ligament repair strategies, particularly for the ACL, which inherently exhibits poor healing capabilities [19,20,26].

Further exploring the EL’s role, Georgiev et al. [27] demonstrated that the EL serves as a reservoir of fibroblasts, progenitor cells, and blood vessels crucial for ligament repair. Their immunohistochemical analyses in the ACL and MCL midsection revealed variable expression levels of vascular endothelial growth factor (VEGF), CD34, and α-smooth muscle actin (α-SMA). While the ACL exhibited slightly higher overall expression of these markers, its limited fibroblast density and reduced collagen expression in the EL hindered its healing capacity. VEGF and CD34, primarily localized in vascular zones, facilitated angiogenesis and vasculogenesis, while α-SMA is associated with myofibroblast activity, aiding structural restoration. These findings underscore the EL’s role in differential ligament healing and highlight the potential for targeted therapies to enhance EL-mediated repair [27].

In addition, Georgiev et al. [28] recently examined the healing differences between the proximal and distal parts of the ACL, further validating the EL theory. Their study found that the proximal EL exhibits higher α-SMA expression, indicating a more significant role in tissue remodeling. In contrast, the distal EL shows elevated CD34 levels, supporting vascularization and progenitor cell activity. Both regions demonstrated significantly higher cell densities compared to the mid-substance of the ACL, suggesting complementary roles in the healing process. These results emphasize the importance of preserving or enhancing EL activity during ACL repair strategies, potentially improving healing outcomes for injuries to this ligament and the better susceptibility to repair of proximal and distal ACL lesions [28].

Similarly, Georgiev et al. [29] investigated the differences in healing capacities between the human knee’s proximal and distal parts of the MCL. The distal EL exhibited higher cell density and greater expression of α-SMA, indicating superior healing potential compared to the proximal part. While CD34 expression was weak in both regions, VEGF was present in the blood vessels without significant regional differences. These findings support the EL theory, highlighting the distal EL’s prominent role in ligament recovery. Targeting the distal EL’s cellular and molecular properties could provide therapeutic benefits for enhancing MCL repair [29]. Table 2 provides a detailed comparison of the structural, cellular, and molecular differences between the EL of the ACL and MCL, focusing on their respective healing capacities. It highlights the distinct regional variations (proximal, mid-substance, and distal) in both ligaments, shedding light on the critical factors influencing healing potential. The table underscores the therapeutic implications of these findings, particularly the potential benefits of targeting the EL to enhance healing outcomes. This comparison offers valuable insights into why the MCL exhibits superior healing compared to the ACL to guide future strategies for ligament repair.

In the current literature, most studies focus on ligament healing in either animal models or human subjects. However, Georgiev and his research team not only investigated the role of the EL in ligament healing but have also advanced EL theory by conducting a comparative analysis across both animal models and translating their findings to humans. Their work provides deeper insights into EL function and its involvement in ligament repair, establishing a new foundation for future research.

## 3. Conclusions

Collectively, the studies discussed highlight the pivotal role of EL in ligament healing, emphasizing its essential contributions to cellular migration, angiogenesis, and collagen synthesis. The EL theory offers a comprehensive framework to understand the distinct healing potentials of the ACL and MCL and the variations within their proximal, midsection, and distal regions. By harnessing the regenerative properties of the EL, targeted therapeutic strategies have the potential to markedly enhance outcomes for ligament injuries, especially for the ACL, which inherently exhibits limited healing capacity.

Despite advancements in understanding the EL theory and its implications for ligament healing, its practical application by morphologists and knee surgeons remains uncertain. Whether this theory will gain widespread acceptance as a key explanation for ACL healing challenges is yet to be determined. Nevertheless, ongoing research should focus on unraveling the molecular and cellular mechanisms of the EL, paving the way for innovative and effective treatments in ligament repair.

## Figures and Tables

**Table 1:** Comparison of the current knowledge and the EL theory on ACL healing failure.

Aspect	Current Knowledge	EL Theory
Structural Characteristics	Focus on ligament ultrastructure differences compared to MCL cells	EL is structurally distinct from the ligament and hypercellular compared to it
Cellular Activity	Limited cellular proliferative capacity in ACL cells	EL contains dynamic fibroblasts producing collagen types I, III, V, and MMPs
Vascularization	Limited blood supply to the ACL after trauma	EL has a significantly higher density of blood vessels than the ligament
Nitric Oxide Levels	Higher levels in the ACL potentially inhibit healing	Not addressed explicitly in the EL theory
Stem Cell Properties	Specific properties of ACL stem cells affect the healing potential	EL contains fibroblasts, fibrocytes, adipocytes, and neurovascular bundles
Matrix Metalloproteinases	Differential expression of MMP-2, MMP-9, and MMP-13	EL fibroblasts dynamically produce MMP-2 and MMP-9
Injury Gap	Cells and blood vessels fail to fill the injury gap adequately	EL penetrates the ligament via the endoligament
Collagen Fiber Properties	Not emphasized	EL consists of small-diameter collagen fibers oriented in various directions.
Location	ACL’s intra-articular position contributes to limited healing	EL is a dynamic structure interacting with the ligament

**Table 2:** Summary of the structural, functional, and immunohistochemical differences between the EL of ACL and MCL.

Feature/Marker	Proximal ACL	Midsubstance ACL	Distal ACL	Proximal MCL	Midsubstance MCL	Distal MCL
Cell Density	Higher than mid-substance, lower than distal	Lowest cell density	Higher than mid-substance, similar to proximal	Lower than distal	Similar to distal	Higher than proximal
Cell Types Present	Fibroblasts, fibrocytes, fewer neurovascular bundles	Fibroblasts, fewer cell types	Fibroblasts, fibrocytes, neurovascular bundles, adipocytes	Fibroblasts, fibrocytes, adipocytes, abundant neurovascular bundles	Similar to distal	Higher fibroblast and neurovascular bundle density
CD34 (Progenitor Cell Marker)	Elevated expression, less prominent than distal	Less prominent expression	High expression, supporting progenitor cell activity	Weak expression	Similar to proximal	Low expression
α-Smooth Muscle Actin (α-SMA)	Higher expression suggests a more significant role in tissue remodeling	Low expression	Elevated expression, supporting tissue remodeling	Low expression	Similar to proximal	Higher expression, aiding remodeling
Collagen Expression	Stronger expression of key collagens (I, III, V)	Weaker expression of key collagens (I, III, V)	Stronger expression of key collagens (I, III, V)	Stronger collagen expression, especially types I, III, V	Similar to distal	Stronger collagen expression, especially types I, III, V
Collagen Fiber Properties	Composed of small-diameter collagen fibers, varied orientations	Smaller diameter, less active remodeling	It is composed of small-diameter collagen fibers oriented in different directions.	Similar to ACL but more active remodeling, enhanced with higher cell density and vascularization	Similar to distal	More active remodeling, greater fiber activity in response to healing
Matrix Metalloproteinase (MMP) Activity	MMP-2 and MMP-9 expression, facilitating remodeling	MMP-2 and MMP-9 expressions are less effective in healing	MMP-2 and MMP-9 expression, aiding in remodeling	Similar to ACL	Similar to distal	Active MMP-2 and MMP-9, aiding superior healing capacity
Vascularization	Limited, less vascularized	Least vascularized	Moderate vascularization, but less than distal	More robust vascularization than proximal	Similar to distal	More robust vascularization than proximal, aiding healing
Vascular Endothelial Growth Factor (VEGF)	Low VEGF expression, less angiogenesis	Limited expression	Higher VEGF expression, aiding angiogenesis and vasculogenesis	VEGF is present but with no significant regional differences	Similar to proximal	VEGF presents enhanced regional blood vessel activity
Functional Role in Healing	Active in remodeling and angiogenesis	Less effective in healing due to low vascularity and cell density	Active in both tissue remodeling and angiogenesis	Prominent in healing through collagen synthesis, fibroblast recruitment, and tissue remodeling	It has a more substantial healing role but is limited compared to distal	The most significant healing role supporting repair through angiogenesis, remodeling, and progenitor cell activation
Overall Healing Potential	Moderate healing aided by collagen and α-SMA	Limited, poor healing potential	High, due to elevated collagen synthesis and cellular activity	Moderate, depends on remodeling activity and fibroblast recruitment	High, yet less superior compared to distal	Excellent, especially for more severe injuries with more active cellular support

## Data Availability

Not applicable.

## Abbreviations

The following abbreviations are used in this manuscript:

EL	Epiligament
ACL	Anterior cruciate ligament
MCL	Medial collateral ligament
MMP	Matrix Metalloproteinase
α-SMA	α-Smooth Muscle Actin
VEGF	Vascular Endothelial Growth Factor
